# Microgynous Queens in the Paleartic Ant, *Manica rubida*: Dispersal Morphs or Social Parasites?

**DOI:** 10.1673/031.010.1701

**Published:** 2010-03-15

**Authors:** Alain Lenoir, Séverine Devers, Philippe Marchand, Christophe Bressac, Riitta Savolainen

**Affiliations:** ^1^ Université François Rabelais, IRBI, UMR CNRS 6035, Faculté des Sciences, Parc de Grandmont, 37200 Tours, France; ^2^ Department of Biosciences, P.O. Box 65, 00014 University of Helsinki, Finland.

**Keywords:** gynes, macrogynes, microgynes

## Abstract

In many ant species, queen size is dimorphic, with small microgynes and large macrogynes, which differ, for example, in size, insemination rate, ovary development, and dispersal tactics. These polymorphic queens often correspond with alternative reproductive strategies. The Palearctic ant, *Manica rubida* (Latreille) (Hymenoptera: Formicidae), lives mostly in mountainous regions in either monogynous colonies, containing one macrogynous queen or polygynous colonies, containing a few large macrogynous queens. In 1998, a colony of *M. rubida* was discovered containing macrogynes and many small alate microgynes that did not engage in a nuptial flight but, instead, stayed in the home nest the following winter. These microgynes were studied more closely by investigating their size, behavior, and spermatheca in relation to *M. rubida* macrogynes and workers. Mitochondrial DNA of macrogynes, microgynes and workers from four nests was sequenced to detect possible genetic differences between them. The microgynes were significantly smaller than the macrogynes, and the head width of the gynes was completely bimodal. The microgynes behaved like workers of the macrogynes in every experiment tested. Furthermore, the microgynes had a normal spermatheca and could be fecundated, but rarely (only one in several years). Finally, all the individuals were genetically identical, except three workers that differed by only one codon position. Because these microgynes have features of both queens and workers, their functional significance in the colony is not yet clear.

## Introduction

In ants, solitary foundation of a new colony generally includes a nuptial flight, followed by dealation and finding a nest site. Solitary colony foundation is risky, and usually more than 99% of virgin winged queens (hereafter referred to as gynes) will die (Hölldobler and Wilson 1990; Wiernasz and Cole 1995). Alternative reproductive strategies have also evolved in ants (Heinze and Tsuji 1995; Peeters and Ito 2001). Fecundated gynes may return to their natal nest or another conspecific nest where they seek adoption. The consequence is a polygynous colony that may form new nests by budding (Bourke and Franks 1995; Peeters and Ito 2001). In polygynous colonies, the gynes tend to be smaller microgynes, rather than the normal macrogynous queens.

In many ant species, queen size is dimorphic, with a large macrogynous morph (usually the normal morph) and a small microgynous morph. The significance of these morphs is rarely understood, but at least three explanations exist. First, the morphs may represent two dispersal tactics with macrogynes dispersing and microgynes filling up the area locally (Hölldobler and Wilson 1977). Some examples include *Myrmica ruginodis* (Elmes 1991), *Ectatomma ruidum* (Lachaud et al. 1999), *Temnothorax rugatulus* (Rüppell et al. 2001) and *Mystrium rogeri* (Molet et al. 2007). Unfortunately, no genetic differences have been searched between these morphs. Second, some microgynes are social parasites of the macrogynes and are genetically differentiated from their hosts. Examples include the microgynes of *Myrmica rubra* (Savolainen and Vepsäläinen 2003) and *Ectatomma tuberculatum* (Hora et al. 2005), now described as a distinct species (Feitosa et al. 2008). Third, the selfish larvae try to develop into gynes when they should become workers with limited or no reproductive capacity (Bourke and Ratnieks 1999; Beekman et al. 2003).

In August 1998, small alate gynes were found in a colony of *Manica rubida* (Latreille) (Hymenoptera: Formicidae) in the French Alps. Typically, the macrogynes of *M. rubida* (total length 10–13 mm) are clearly bigger than the workers (6–8 mm) (Bernard 1968), but the gynes discovered were the same size as the workers. Thus these gynes were microgynes, and, apparently, the discovery was novel, as we could not find in the literature any information on microgynes of *M. rubida*. Later, more colonies with microgynes were found in the same area.

Because *Monica* is the sister genus to *Myrmica* (Bolton 2003), some similarities in the microgynes of *M. rubida* and either *Myrmica ruginodis* or *Myrmica rubra* were expected. Thus, the *M. rubida* microgynes were studied by investigating their size, behavior and spermatheca in relation to *M. rubida* macrogynes and workers. Mitochondrial DNA of the microgynes and macrogynes was also sequenced to detect possible genetic differences between them.

## Materials and Methods

*Manica rubida* is frequent in European mountain regions from 500 to 2000 MASL (Seifert 1996, 2007) and in France in the Alps and Central Massif between 700 and 1800 MASL. It nests in open fields with low slopes where it constructs large, but superficial, subterranean nests (Bernard 1968). Colonies contained several thousand workers and were either monogynous having a single queen, or polygynous with a few large, macrogynous queens (Cammaerts and Cammaerts 1987). Colony foundation is semi-claustral and takes place after a nuptial flight (Le Masne and Bonavita 1969).

*Manica rubida* was studied in the Giffre Valley (Haute-Savoie, French Alps) that runs east-west from Taninges (600 MASL, 46° 06.37 N, 6° 33.30 E) and covers about 10 km, ending in a large, touristic area (Cirque du Fer à Cheval, 1080 MASL, 46° 03.36 N, 6° 47.29 E). *Manica rubida* was frequent on the flat banks of the river that had little vegetation, composed mainly of alder, *Alnus sp*.. Other ant species in the area were *Formica selysi* and *F. lemani*. The first *M. rubida* colony (colony or nest A) with both macrogynes and microgynes was observed in August 1998, near Samoëns (698 MASL, 46° 04.39 N, 6° 42.43 E). This colony had been observed regularly for many years prior to 1998 without the sighting of any microgynes. Thereafter, this colony was monitored at least three times per year, particularly after the nuptial flight period at the end of May/early June and after winter, to assess the presence of alate microgynes in the nest.

In July 2002, a small nest (colony A_1_) was observed 5 m from colony A, which suggested budding from nest A. In June 2003, another colony (H) with microgynes was observed three km east of nest A, in Morillon (46° 05.14 N, 6° 41.18 E). In June 2004, 15 colonies along an area of 6 km were found between La Rivière Enverse and Samoëns, with some colonies having both macrogynes and microgynes and some containing only macrogynes. In 2005, a large colony (P) was found in Verchaix (46° 05.33 N, 6° 39.54 E) with only microgynes. *Manica rubida* was searched for in a few other places: Bessans (Savoie, 1500 MASL, 45° 19.11 N, 7° 01.24 E) and Urle (Vercors, Isère, 1434 MASL, 44° 53.51 N, 5° 19.16 E), where no colonies with microgynes were found. In August 2006, one colony with both macrogynes and microgynes was discovered in the Hautes-Alpes, La Chapelle en Valgaudemar (1180 MASL, 44° 49.47–48 N, 6° 14.00–21 E), in a place where they were absent some years before.

### Size of gynes and males

The maximum head width, maximum thorax width and thorax length of the macrogynes (*n* = 35) and microgynes (*n* = 55) of colony A were measured using a binocular microscope. The HW of males were also measured, including the eyes, from two colonies with only macrogynes (*n* = 45) and two colonies with only microgynes (*n* = 41).

### Aggression tests

In the field, antagonistic behavior occurs between workers of different *Monica* nests (Cammaerts and Cammaerts 1987; A. Lenoir personal observation). In fissioning species, workers of recently separated nests, however, often interact with workers of their mother nest and thus are not aggressive to each other (Ichinose et al. 2005). To investigate whether nest A_1_ originated (fissioned) from nest A, aggression tests were conducted between the workers of colonies A and A_1_. Several hundred foragers of colony A were collected, as was the entire colony A_1_, which contained one dealate microgyne and several hundred workers. Control experiments were set-up between workers of the same nest (nests H and M). If A_1_ originated from nest A, aggression should be weak between their workers. As aggression increases with geographical distance between nests (Cammaerts and Cammaerts 1987), aggression was compared between workers of nests A and A_1_ and those of nests H and M, which were separated by several km from the former nests.

First three workers (residents) of one nest were placed on an arena (diameter = 85 mm, walls coated with fluon to prevent escaping); then a marked individual (intruder) from another nest was added. The intruder was placed in a tube in the middle of the arena, and after one min, the tube was removed . The bottom of the arena was covered with filter paper that had been kept for several hours in the nest of the three resident ants to impregnate their colony odor on it (Cammaerts and Cammaerts 2000). The behavior (see below) of the intruder was recorded every 5 s for 5 min. Old workers (darker color) were selected for the tests, as they are considered more aggressive than young ones (Cammaerts-Tricot 1974). A global aggression index, AI, was calculated according to a previously published formula (Hefetz et al. 1996):

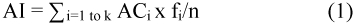

AC is a coefficient of aggression for each behavior, f is its frequency, and n s the total number of acts, i. The aggression coefficients for each act were: 1 = threat (opening of mandibles); 2 = biting; 3 = stinging or its trial; 0 = all other behaviors. To consider the possible differences of aggression between colonies, one intruder A was tested against three B resident workers, and, reciprocally, one B intruder was tested against three residents of A. For each situation, 20 tests were carried out. The data was analyzed with the Kruskal-Wallis or Mann-Whitney test.

In founding colonies, the first workers are generally small and “nanitic” (Porter and Tschinkel 1986). Therefore, to check if nest A_1_contained these nanitic workers, the size of workers from nest A_1_ (*n* = 50) were measured, as were workers from another nest that contained only macrogynes (*n* = 51). The size index (AI) was used to weigh the head and thorax equally (Rüppell et al. 1998):

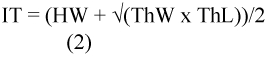

Where IT is size index, HW is head width, ThW is thorax width and ThL is thorax length.

### Colony tasks: Egg-laying, brood retrieval, division of labor

As microgynes stayed in their natal nest and did not engage in nuptial flight, whether they exhibited any gyne or worker behaviors was tested. To compare egg-laying rate between macrogynes and microgynes, six combinations of individuals collected in the field were formed: one dealated microgyne alone (*n* = 10), and with 20 workers (*n* = 12); one alate microgyne alone (*n* = 21), and with 20 workers (*n* = 6); groups of 20 workers (*n* = 10); and one macrogyne with 20 workers (*n* = 7). These groups were reared in the laboratory, and all eggs laid were counted twice a week for two months.

In preliminary experiments, in groups of workers with one macrogyne, only workers retrieved brood to the nest. To further investigate whether microgynes and macrogynes differed in their behavior, two experiments were performed. (1) In broodretrieval experiments, a group of 20 workers and 10 microgynes (*n* = 13), were placed one group at a time in a glass tube with some water plugged with cotton, and the tube was closed with cotton (the ants and brood were used only once). A black cover was placed over the tube to represent the conditions inside a nest, and the tube was placed in a box (10 × 15 cm). Then, 10 larvae and 10 pupae were deposited near the entrance of the tube, and the cotton plug was removed. The total time to retrieve all the brood was recorded from the first contact with the brood. (2) In the division of labor experiments, groups of 20 individuals with 20 larvae and 20 pupae were placed in a plaster nest covered with a piece of glass to allow observation of ants. Three nests were made: 10 alate microgynes and 10 workers, 10 dealate microgynes and 10 workers, and one control with 20 workers. Each individual was marked with a unique combination of color dots. The nest was linked to the arena (as above) where food was deposited. For each nest, the behavior of each individual (scan sampling) was recorded instantaneously in at least 5 min intervals on three days (*n* = 100).

### Foraging in the field

Dealate macrogynes were observed foraging in the field in the summer, thus verifying their non-claustral colony foundation (Le Masne and Bonavita 1969). In the territories of established colonies, foraging alate microgynes were also repeatedly observed each summer. In one nest (M) in June 2004, the microgynes were offered cookie crumbs 80 cm from their nest entrance. All foraging microgynes were marked with a dot of paint, and the number of different microgynes with cookie crumbs was counted for one hour.

### Spermatheca and spermatozoid count

To observe the status of their spermatheca, 50 gynes (3 alate and 11 dealate macrogynes, 26 alate and 10 dealated microgynes) were dissected and their sperm counted according to Lenoir et al. (1999). Spermathecae were isolated in a saline drop (128.3 m*M* NaCl, 4.7 m*M* KCl, 2.3 m*M* CaCl_2_) and then transferred to 100 µl of the same saline solution. They were opened with forceps. The resulting suspension was gently shaken to disperse sperm and then homogenised. Three 1-µl drops of the final suspension were deposited on a clean microscope slide and air dried. The preparations were fixed by ethanol, dried again, and incubated for 10 min in a 2 µg/ml DAPI solution (4-6-diamidino-2-phenylindole) to stain nuclei. All sperm were counted in the three drops under a fluorescence microscope to obtain the total amount of sperm stored in the spermatheca.

### Genetic analysis

DNA was extracted from six individuals from four nests (in total, 24 individuals): three workers (presumed macrogynes, because no macrogynes were available) and three microgynes from colony A_1_, and two macrogynes, microgynes and workers from colonies H, J5 and M. Partial cytochrome c oxidase subunit I gene was amplified using the primers C1-J-1751 (alias Ron), C1-J-2183 (alias Jerry) and TL2-N-3014T (alias Pat) (Simon et al. 1994), following the molecular methodology of Savolainen and Vepsäläinen (2003). We edited and aligned the sequences and visualized their base pair differences using Sequencher v. 4.7. (Gene Codes, www.genecodes.com).

## Results

All field observations since 1998 indicated that microgynes did not engage in nuptial flights, but, instead, overwintered in their natal nest. They kept their wings until the next spring which damaged them such that in spring some of the microgynes were dealated. It is unknown if microgynes survive more than one year.

### Size of gynes and males

**Figure 1:**
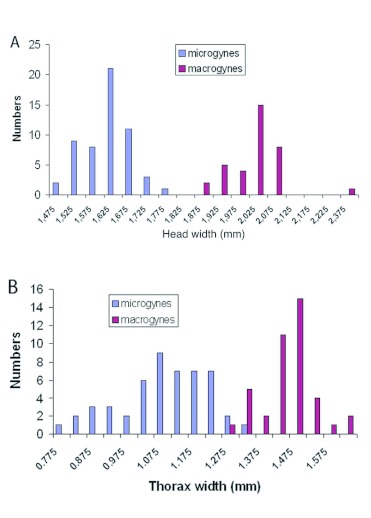
Distribution of maximum width of the head (A) and the thorax (B) of *Manica rubida* gynes. The intermediate individuals in B were classified as micro or macrogynes according to their head width. High quality figures are available online.

**Figure 2:**
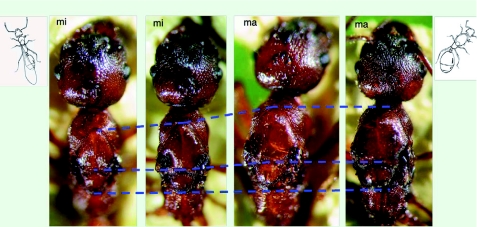
Thorax of *Manica rubida* gynes. On the left the thorax of two microgynes (mi); on the right, the thorax of 2 macrogynes (ma). High quality figures are available online.

The head width of the macrogynes and microgynes was clearly dimorphic without overlap (Figure 1A). The head width of the macrogynes was 2.02 mm ± 0.085 (*n* = 35), and that of the microgynes was 1.61 mm ± 0.065 (*n* = 55) (Student t-test, p = 0.001). The thorax width was also bimodally distributed, though with some overlap (Figure 1B, 1.45 mm ± 0.077, *n* = 41 vs. 1.08 mm ± 0.130, *n* = 50; Student t-test, p = 0.0009). The scutum and scutellum were more developed in the macrogynes than in the microgynes. The dotted lines indicate that the scutum increases in size in microgynes (not measured) (Figure 2). The size of males was not different between the colonies producing either macrogynes or microgynes (1.04 mm ± 0.15, *n* = 44 vs. 1.06 ± 0.13, *n* = 45; Student t-test, p = 0.65, for macrogynes and microgynes, respectively). In contrast, the size of the males was dependant of the colony. For the males in the two microgyne colonies, the size was 0.94 mm ± 0.11 (*n* = 14) and 1.12 mm ± 0.09 (*n* = 30) (Student t-test, p < 0.0001). For the two macrogyne colonies, the size was 0.95 mm ± 0.14 (*n* = 20) and 1.12 mm ± 0.11 (*n* = 25) (Student t-test, p < 0.0001).

### Aggression tests

**Figure 3:**
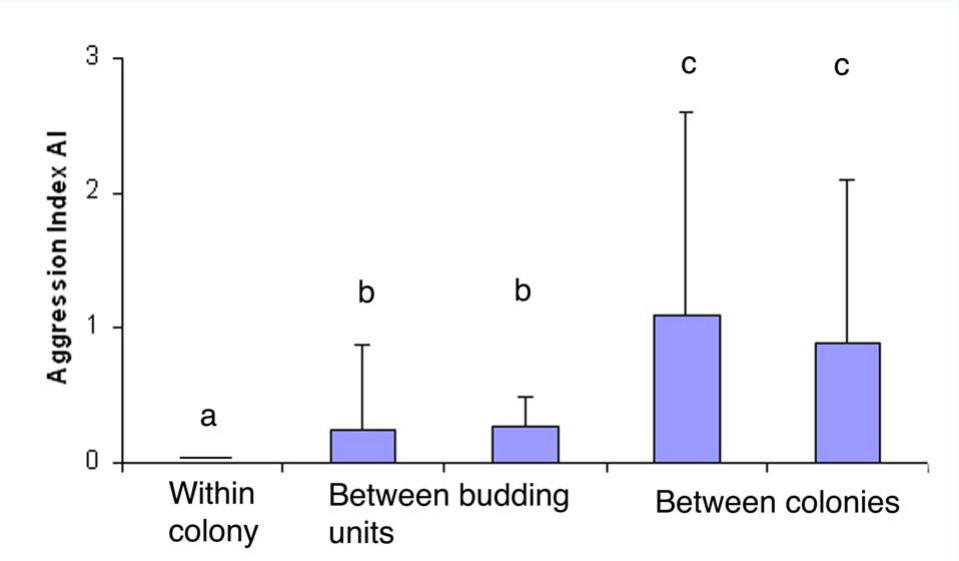
Aggression Index (mean ± SD) between 3 workers and one intruder (and reverse) from the same colony (within colony); from nests A and A_1_ (between budding units); from foreign nests (between colonies). Different letters indicate significant differences. *n* = 20 for each case. High quality figures are available online.

**Figure 4:**
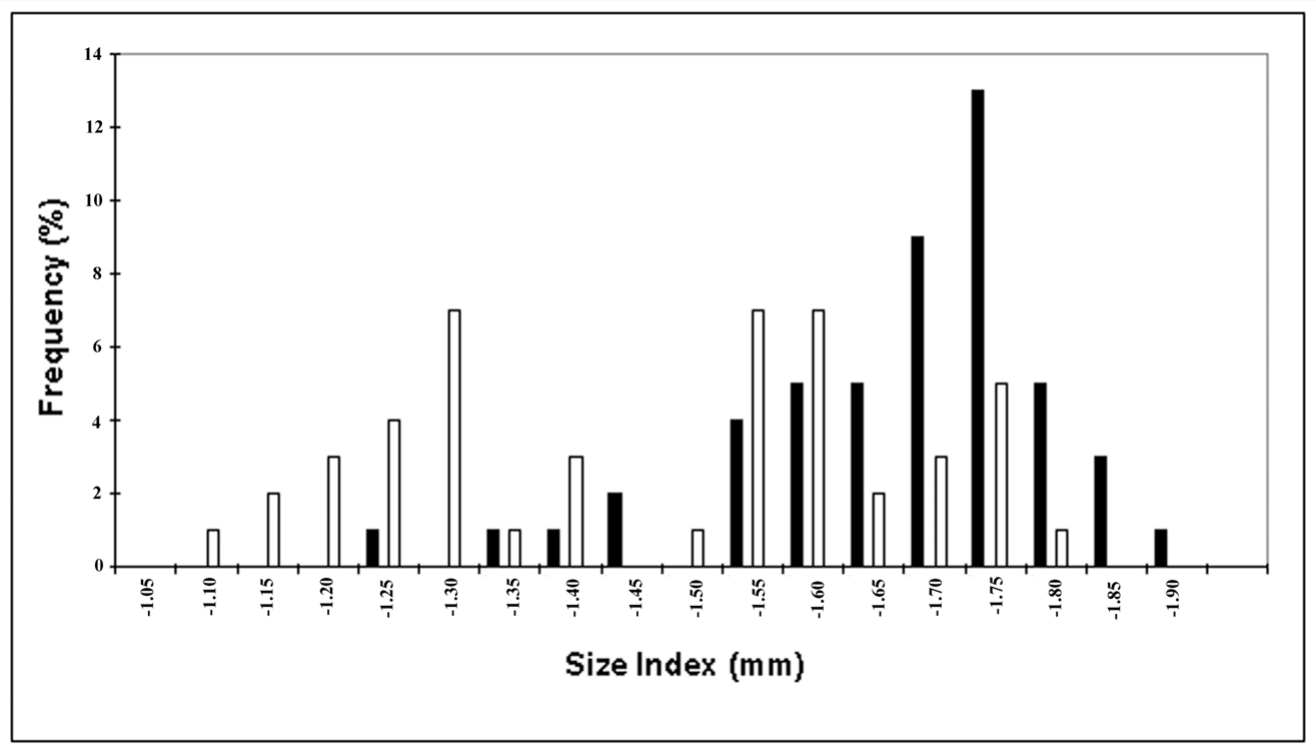
Size index distribution for workers of nest A_1_ (white) and nest C (black), with only *Manica rubida* macrogynes. High quality figures are available online.

Intracolonial aggression was absent in the control tests (AI within colony = 0; Figure 3). The workers of nest A_1_ were smaller than those of nest C (Student t-test, p < 0.001). The workers of nest A_1_ included two size classes (mean index IT 1.3 and 1.6), whereas the workers of nest C, a typical nest with macrogynes, were bigger (IT 1.75) (Figure 4). These data are congruent with the behavioral data, indicating that the budded nest is composed of two worker types: small workers and large workers.

### Colony tasks: Egg-laying, brood retrieval and division of labor

Among the 6 types of experimental colonies followed during two months, neither workers nor non-inseminated microgynes (winged or wingless) laid eggs, whereas macrogynes normally laid eggs and produced workers.

Macrogynes did not engage in brood rescue. In contrast, microgynes were efficient in brood-retrieval tests; there was no difference between microgynes (318 ± 126 s) and workers (332 ± 210 s) in mean retrieval time (Wilcoxon test, p = 0.361, *n* = 13).

In the laboratory, microgynes behaved like workers. They spent on average 10 to 25% of their time in caring for the brood (no significant differences between workers and microgynes in the three sets). Occasionally, a worker ant tried to cut the wings of a microgyne, but again, both workers and microgynes practiced this behavior. Behaviors that distinguished microgynes from workers were not detected.

### Foraging in the field

Observations confirmed that dealate macrogynes (founding queens) foraged solitarily during their non-claustral founding period in summer. They foraged only in areas unoccupied by *Manica* colonies, and they never foraged in *Manica* territories. In mature colonies producing microgynes, some of them left the nest to forage, and they behaved like foragers, retrieving food items that were brought to the nest. In one hour, there were 53 different microgynes foraging around the same nest, which is equivalent to the foraging task of a worker.

### Spermatheca and spermatozoid count

**Figure 5:**
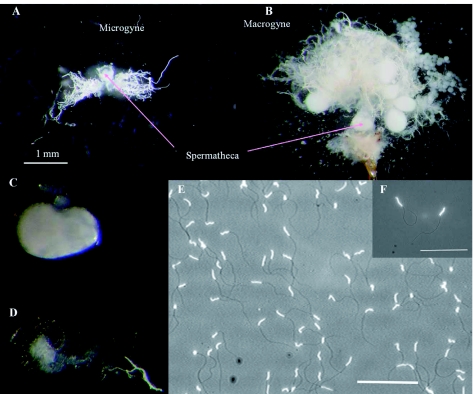
Spermatheca and ovaries of *Manica rubida* gynes. A) empty spermatheca and non-developed ovarioles of a microgyne. B), full spermatheca and functional ovary of a macrogyne. Scale = 1 mm. Spermatheca of a fecundated macrogyne (C) and an empty one from non-fecundated microgyne (D). E) Non diluted sperm of *M. rubida* stained with DAPI. Scale = 100 µ. F) detail of spermatozoids. Scale = 100 µ. High quality figures are available online.

The microgynes had normal spermatheca (Figure 5A–D), but only 1 of 38 dissected gynes (2.8%) was inseminated. All dealated macrogynes were inseminated. The sperm counts of macrogynes were 340,388 ± 50,520 (*n* = 17) (see spermatozoids in Figure 5E, F). The single inseminated microgyne contained spermatozoids, but unfortunately the sperm could not be counted.

### Mitochondrial DNA differences

For all 24 individuals, 1023 bp were obtained (Genbank accession numbers EU864121– EU864144). Only three haplotypes were found; all individuals were genetically identical, except for three workers. The two workers of nest J5 had a substitution at one third-codon position (C at position 492), and one worker of nest A_1_ had a substitution at another third-codon position (C at position 594). The other individuals had T in these positions.

## Discussion

The size distribution of *Manica rubida* gynes was clearly bimodal and not a result of random phenotypic variation. The males, however, were not dimorphic. Because these microgynes had features of both the queens (morphology, possibility to be inseminated) and workers (brood retrieval, foraging), their functional significance in the colony is not clear. There are at least four hypotheses for the existence of microgynes in ant colonies.

These hypotheses are not mutually exclusive and combine proximate (hypothesis 1) and ultimate explanations (hypothesis 2–4). (1) Numerous studies have accumulated evidence that differences in larval environment, particularly in nutrition, determine the developmental path of a larva into a reproductive worker or a gyne (Wilson 1971; Hölldobler and Wilson 1990). Recent work on caste determination indicates, however, that it is, instead, genetically based, at least in *Pogonomyrmex* (Julian et al. 2002; Volny and Gordon 2002; Helms Cahan and Keller 2003) and *Reticulitermes* (Hayashi et al. 2007). Therefore, a mutation of developmental genes may have appeared, preventing larvae from developing into normal macrogynes. (2) Microgynes are a consequence of a caste conflict between larvae and the queen or workers. The selfish larvae try to escape queen or worker control over sexual production and develop into gynes rather than becoming workers with limited reproductive capacity. This is especially true for *Manica* workers, which are completely sterile. The brood of social Hymenoptera is generally considered to have little ability to affect the choice of their development into sexuals or workers, because they are fed by adult workers (Bourke and Ratnieks 1999; Beekman et al. 2003). When larvae are reared in sealed cells, like in *Melipona* bees, they may choose to develop into workers or queens (Ratnieks 2001). This may also take place in ant species with larvae that feed themselves. With a limited amount of food, miniaturization is one mechanism for diploid larvae to become queens (Ratnieks et al. 2006). According these authors, it is the most likely explanation for the existence of microgynes in social Hymenoptera. (3) The microgynes are social parasites of the macrogynes and genetically differentiated from their hosts. Examples include the microgynes of *Myrmica rubra* (Savolainen and Vepsäläinen 2003) and *Ectatomma tuberculatum* (Hora et al. 2005), now described as a distinct species, *Ectatomma parasiticum* (Feitosa et al. 2008). In *M. rubra*, the microgynous social parasite differs genetically, though only slightly, from its host (Savolainen and Vepsäläinen 2003). These authors suggested that this social parasite/host pair may represent an incipient sympatric speciation process taking place through intraspecific social parasitism. Sympatric speciation has been a highly debated process, but now there are well documented cases of it, for example, in palm trees (Savolainen et al. 2006). In *M. rubida*, mitochondrial DNA analysis showed only minor genetic differences: three workers (presumed issued from macrogynes) differed in one base pair from microgynes and macrogynes. Further genetic analyses are needed, but it seems very improbable they can be considered as two species. (4) The microgynes represent an alternative reproductive tactic and are part of a dispersal strategy. The small microgynes may spread locally after intranidal copulation from the mother nest, whereas the large macrogynes establish new colonies after a nuptial flight (Hölldobler and Wilson 1977). Some examples include *Myrmica ruginodis* (Elmes 1991), *Ectatomma ruidum* (Lachaud et al. 1999), *Temnothorax rugatulus* (Rüppell et al. 2001) and *Mystrium rogeri* (Molet et al. 2007). The presumed dispersal strategy of *M. rubida* is not efficient, however, as the colonies produce many microgynes that stay in the home nest as workers and forage. Foraging of *Manica* macrogynes is normal during the non-claustral founding stage, but it has never been observed in queens after colony foundation.

The cost of producing numerous microgynes in *M. rubida* is also worth discussing. If microgynes behave like workers and are of the same size as the workers, there is presumably no cost. If the microgynes work and reproduce less than the workers, however, then the colony may suffer some costs. This may be a case where an individual's interest (to become a queen instead of a worker) conflicts with the colony interest (to maximize the colony productivity). The conflict may be unstable and selected against, like semi-claustral foundation (Peeters and Ito 2001).

## Abbreviation

MASL: meters above sea level,
gynes: virgin winged queens
